# Colistin causes profound morphological alteration but minimal cytoplasmic membrane perforation in populations of *Escherichia coli* and *Pseudomonas aeruginosa*

**DOI:** 10.1007/s00203-018-1485-3

**Published:** 2018-02-08

**Authors:** Noëlle H. O’Driscoll, T. P. Tim Cushnie, Kerr H. Matthews, Andrew J. Lamb

**Affiliations:** 1 School of Pharmacy and Life Sciences, Robert Gordon University, Sir Ian Wood Building, Garthdee Road, Aberdeen, AB10 7GJ UK; 2 Faculty of Medicine, Mahasarakham University, Khamriang, Kantarawichai, Maha Sarakham 44150 Thailand; 3 Graduate School, Robert Gordon University, Health and Social Care Building, Garthdee Road, Aberdeen, AB10 7QG UK

**Keywords:** Colistin, Polymyxin E, Mechanism of action, Mode of action, Flow cytometry, SEM

## Abstract

Whilst colistin (polymyxin E) represents the last mainstream treatment option for multidrug-resistant Gram-negative pathogens, details of its mechanism of action remain to be fully resolved. In this study, the effects of sub-inhibitory, inhibitory-bactericidal, and supra-bactericidal levels of colistin on the membrane integrity and morphology of *Escherichia coli* and *Pseudomonas aeruginosa* were investigated using potassium loss, flow cytometry, and scanning electron microscopy (SEM). Supra-bactericidal colistin concentrations induced just 4–12% intracellular potassium loss from bacteria after 24 h. Flow cytometry data suggested colistin might alter cell arrangement, and SEM confirmed the antibiotic causes bacterial aggregation. Filamentation was not detected in either species at any concentration or time-point up to 24 h. These results argue against the hypotheses that colistin kills bacteria by puncturing the cytoplasmic membrane or disrupting DNA synthesis. The colistin-induced bacterial aggregation detected has implications for the interpretation of MBC, time-kill, and other test results obtained with this antibiotic.

**Electronic supplementary material:**

The online version of this article (10.1007/s00203-018-1485-3) contains supplementary material, which is available to authorized users.

## Introduction

Colistin (polymyxin E) is an antibiotic with a spectrum of activity that includes problematic carbapenem-resistant and extensively drug-resistant Gram-negative bacteria such as *Pseudomonas aeruginosa* and *Escherichia coli*. Systemic use of colistin largely ceased in the 1970s due to concerns about nephrotoxicity and neurotoxicity, but growing resistance to other antibiotic classes prompted a reassessment of its safety at the turn of the century (Tängdén and Giske 2015; Tran et al. 2016). Under suitable dosage regimens and with careful monitoring, it is now accepted that the risk of colistin-induced kidney or nerve damage can be minimized (Kelesidis and Falagas 2015; Shields et al. 2017), and the antibiotic has been returned to use as a last option or salvage therapy (Poirel et al. 2017). Clinical indications include treatment of ventilator-associated pneumonia and lung infections in cystic fibrosis patients (Gu et al. 2014; Liu et al. 2015) as well as bacteraemia and urinary tract infections (Tängdén and Giske 2015; Bader et al. 2017) caused by extensively drug resistant Gram-negative organisms. Transmissible colistin resistance emerged in 2011 and has spread worldwide, but its prevalence is still quite low (Giske 2015; Liu et al. 2016; Baron et al. 2016).

Because colistin is rapidly bactericidal against Gram-negative but not Gram-positive species, and has a strong binding affinity for lipopolysaccharide (LPS), it is widely accepted that this antibiotic targets the bacterial outer membrane. However, precise details of how colistin exerts its antibacterial effect remain unclear (Tran et al. 2016; Poirel et al. 2017). At the time of its discovery and development in the late 1940s, regulatory and licensing bodies such as the Medicines and Healthcare products Regulatory Agency (MHRA) were not in existence, and the substantial body of microbiological and pharmacological evidence now necessary for licensing an antibiotic was simply not required (Landersdorfer and Nation 2015). Therefore, a relatively limited body of information on the antibacterial effects of colistin is available to inform rational use (Honoré et al. 2014; Theuretzbacher 2014). Elucidation of further details could permit optimization of colistin dosing, prediction of synergistic drug combinations, prediction of drug combinations that reduce the risk of additional resistance emerging, and the development of second-generation polymyxins (Deris et al. 2014; Poirel et al. 2017).

Several recent studies have explored how colistin interacts with the bacterial cell using transmission electron microscopy (TEM), model membranes, and other techniques. Consensus exists that initial electrostatic and hydrophobic interactions occur between colistin molecules, which are positively charged, and the negatively charged LPS layer of the outer membrane. This first step, sometimes referred to as ‘self-promoted uptake’, leads to displacement of the divalent cations (Ca^2+^ and Mg^2+^) that normally stabilize the LPS monolayer (Velkov et al. 2013). How colistin then kills bacterial cells is uncertain. One longstanding hypothesis is that colistin molecules disrupt the physical integrity of the cytoplasmic membrane, causing leakage of intracellular contents (Velkov et al. 2010; Poirel et al. 2017). Another hypothesis is that colistin causes the inner layer of the outer membrane and outer layer of the cytoplasmic membrane to come together, resulting in phospholipid exchange, and an osmotic imbalance (Clausell et al. 2003; Velkov et al. 2013). Additional possibilities, not necessarily mutually exclusive, are that colistin inhibits vital respiratory enzymes (type II NADH-quinone oxidoreductases) at the cytoplasmic membrane (Deris et al. 2014), that colistin induces the formation of reactive oxygen species when it crosses the cytoplasmic membrane (Yu et al. 2017) or, in a similar manner to other cationic antimicrobial peptides, that colistin binds to bacterial DNA (Kong et al. 2011) inhibiting replication and transcription.

The purpose of the present study was to gain further insight into the interaction between colistin and bacterial cells including its underlying mechanism of action. Cytoplasmic membrane damage was assessed by measuring potassium loss from colistin-treated cells, as leakage of this intracellular solute is an early sign that membrane integrity has been compromised (Yu et al. 2016; Liang et al. 2016). Colistin-treated bacteria were also examined by flow cytometry and scanning electron microscopy (SEM) as changes in the size and shape of cells can be useful in identifying antibiotic targets too (Peach et al. 2013; Cushnie et al. 2016). To account for the fact that antibiotic-induced morphological changes can vary with antibiotic concentration (Alsteens et al. 2008), inoculum density (Diver and Wise 1986), and test bacterial species (Wojnicz et al. 2007), we examined the effects of sub-inhibitory, inhibitory, and bactericidal concentrations of colistin upon two species, *E. coli* and *P. aeruginosa*, using minimum inhibitory concentration (MIC) and minimum bactericidal concentration (MBC) values determined for assay-specific inoculum densities.

## Materials and methods

### Bacteria

*E. coli* NCTC 4174, *P. aeruginosa* NCTC 6750 and *Staphylococcus aureus* NCTC 6571 were obtained from the National Collection of Type Cultures (Health Protection Agency Culture Collections, UK). These bacteria were stored, sub-cultured, harvested, and washed as described previously (O’Driscoll et al. 2013).

### Antibacterial agents, chemical reagents, and growth media

Colistin sulfate, phosphate buffered saline (PBS) tablets, isopropanol (99+%), and sodium phosphate were purchased from Sigma-Aldrich Company (Poole, UK). Potassium dihydrogen orthophosphate SLR and glutaraldehyde were from Fisons Scientific (Loughborough, UK), and sodium carbonate was from BDH (Poole, UK). Sodium chloride (NaCl; general-purpose grade), acetone (technical grade), and formaldehyde were purchased from Fisher Scientific (Loughborough, UK), and nutrient broth and agar were from Oxoid (Basingstoke, UK). The Bac*Light*™ Live/Dead Kit was obtained from Molecular Probes (Invitrogen, Paisley, UK), while flow cytometry Sheath fluid, Flow Check™ fluorospheres and all plastic disposable equipment were from Beckman Coulter (Buckinghamshire, UK).

### Determination of MIC and MBC values for colistin

MICs were determined according to the CLSI broth microdilution method (CLSI 2006; Wiegand et al. 2008), but with the following necessary modifications. Rather than using the CLSI-recommended inoculum density of 5 × 10^5^ cfu mL^−1^ and CLSI-recommended incubation time of 16–20 h, MICs were determined for assay-specific inoculum densities (1 × 10^6^ cfu mL^−1^ bacteria for the potassium loss and flow cytometry assays, and 1 × 10^7^ cfu mL^−1^ bacteria for scanning electron microscopy analysis) and assay-specific incubation time of 24 h. Microtitre plates (96-well; Bibby Sterilin, Staffordshire, UK) were sealed with an optically clear, gas-permeable seal (Fisher Scientific) and incubated at 37 °C (Versamax Microplate Reader, Molecular Devices, Berkshire, UK).

MBC values were established by a replica plating procedure, with 1–2 µL from each well transferred to the surface of a 13.5-cm-diameter nutrient agar plate using a 96-pin multi-point replicator (Boekel Scientific, US). The presence or absence of growth was recorded after 24 h aerobic incubation at 37 °C. All assays were conducted in triplicate on six separate occasions.

### Quantification of intracellular potassium loss from populations of colistin-treated bacteria

Populations of 1 × 10^6^ cfu mL^−1^ *E. coli* and *P. aeruginosa* were incubated with inhibitory-bactericidal (1 × MIC and 1 × MBC) and supra-bactericidal (10 × MBC) levels of colistin (37 °C; 150 rpm) for various time periods (0, 1, 2, 4, 8 and 24 h), and examined for potassium loss using a flame atomic absorption spectrophotometer (Model AA3110, Perkin Elmer, Beaconsfield, UK) as described previously (O’Driscoll et al. 2013). Untreated cells were used as a negative control. Sub-inhibitory levels of colistin (1/20 × MIC) induced negligible potassium loss during preliminary testing, and were excluded from the study to allow more rapid processing of samples from the other test conditions (i.e. untreated, inhibitory-bactericidal, and supra-bactericidal). Total intracellular potassium content was determined by sonicating each cell sample (Misonix XL 2010 Ultrasonic Liquid Processor; Heat Systems, Farmingdale, US) at 20 kHz for four 30-s pulses, pausing between pulses to place the centrifuge tube on ice. All assays were conducted in triplicate on three separate occasions.

### Analysis of colistin-treated bacterial populations by flow cytometry

Populations of 1 × 10^6^ cfu mL^−1^ *E. coli* and *P. aeruginosa* were incubated in nutrient broth containing sub-inhibitory (1/20 × MIC), inhibitory-bactericidal (1 × MIC and 1 × MBC) and supra-bactericidal (10 × MBC) levels of colistin (37 °C for 24 h; 150 rpm) prior to analysis by flow cytometry using Bac*Light*™ Live/Dead Bacterial Viability reagents (Invitrogen). In accordance with the manufacturer’s instructions, bacterial populations incubated in aqueous 0.9% (w/v) NaCl and 70% (v/v) isopropyl alcohol were employed as viable and non-viable controls. All bacterial populations, both control and colistin-treated, were prepared for flow cytometric analysis as follows. Firstly, 10 µL of each bacterial suspension was pipetted into a series of sterile 1.5-mL microcentrifuge tubes containing 987 µL of 0.9% (w/v) NaCl with 1.5 µL of Syto9 and 1.5 µL propidium iodide (PI) fluorescent dye solutions. Tubes were then incubated in the dark for 15 min at room temperature prior to analysis using an Epics®XL MCL flow cytometer (Beckman Coulter). Prior to using the flow cytometer, the discriminator was set at 0.2 µm and the laser alignment was checked and adjusted using Flow Check™ fluorospheres. The test samples were processed once the half-peak co-efficient variation was ≤ 2% on channels FL1, FL2, FL3 and FL4. Channels FL1 and FL3 were set to detect Syto9 and PI, respectively. When being analysed, each sample either had 10,000 events recorded requiring approximately 5 s for controls, or was left for up to 5 min permitting the maximum number of events to be gathered. All assays were conducted in triplicate on three separate occasions.

### Examination of the effect of colistin treatment on bacterial cells and populations by scanning electron microscopy

Populations of 1 × 10^7^ cfu mL^−1^ *E. coli* and *P. aeruginosa* were incubated with sub-inhibitory (1/20 × MIC), inhibitory-bactericidal (1 × MIC and 1 × MBC), and supra-bactericidal (10 × MBC) levels of colistin (37 °C; 150 rpm) for various time periods (0, 1, 2, 4, 8, and 24 h), then prepared for and analysed by SEM (LEO S430, Carl Zeiss SMT Ltd., UK) using a protocol described previously (O’Driscoll et al. 2013). A slightly higher bacterial cell density was needed in this assay compared to potassium loss and flow cytometry assays, as cells in untreated control populations of 1 × 10^6^ cfu mL^− 1^ appear sparse when examined by SEM prior to 24 h incubation (data not shown) and antibiotic-induced morphological changes are known to occur much earlier than 24 h (sometimes within just 1 h of treatment; Cushnie et al. 2016). The size of cells and cell aggregates was determined using LEO S430 software. After extensive examination at a range of magnifications (4000× to 400,000×), micrographs of those fields of view representing typical bacterial characteristics were captured.

## Results

### Determination of MIC and MBC values for colistin

The MIC and MBC values determined for colistin against the test species of bacteria, at cell densities of 1 × 10^6^ and 1 × 10^7^ cfu mL^−1^, are shown in Table 1. These results correspond well with previously published MICs and MBCs (Li et al. 2006; Cummins et al. 2009; Alhanout et al. 2010), and confirm the lack of activity of colistin against Gram-positive bacteria.

**Table 1:** Minimum inhibitory concentration (MIC) and minimum bactericidal concentration (MBC) values of colistin against different cell densities of the test bacteria

Strain	MIC (mg L^−1^)		MBC (mg L^−1^)	
	1 × 10^6^ cfu mL^−1^	1 × 10^7^ cfu mL^−1^	1 × 10^6^ cfu mL^−1^	1 × 10^7^ cfu mL^−1^
*S. aureus* NCTC 6571	100	156	100	156
*E. coli* NCTC 4174	0.28	9.8	0.28	9.8
*P. aeruginosa* NCTC 6750	1.5	4.9	1.5	4.9

### Quantification of intracellular potassium loss from populations of colistin-treated bacteria

**Fig. 1:**
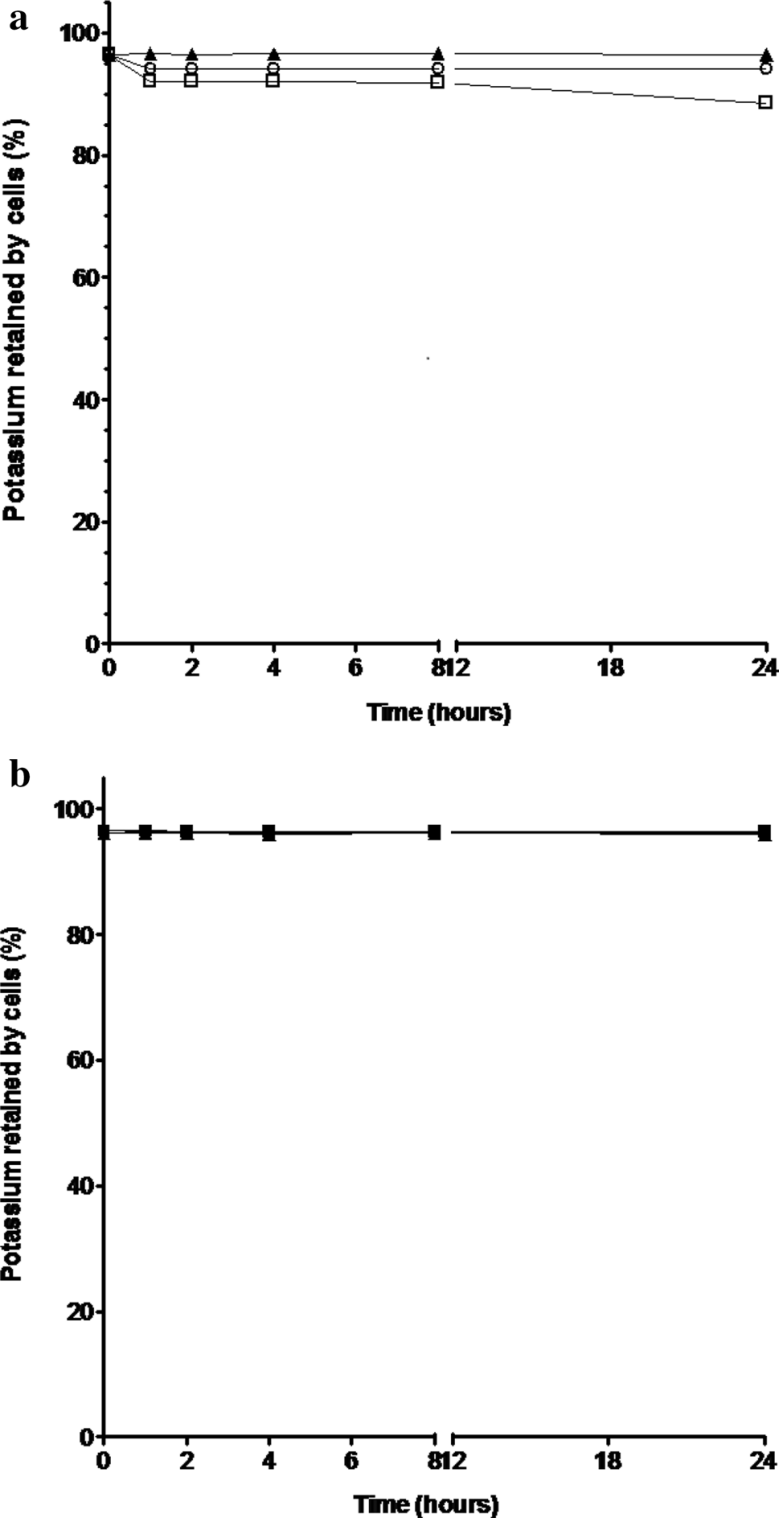
Potassium loss from populations of **a** 1 × 10^6^ cfu mL^−1^ *E. coli* and **b** 1 × 10^6^ cfu mL^−1^ *P. aeruginosa* incubated with and without colistin for 24 h (error bars represent standard error of the mean for three assays). The total intracellular potassium content, determined by sonication, was 3.2 mg/L for both *E. coli* and *P. aeruginosa*. Triangle: untreated control; unfilled circle: 1 × MIC (equal to 1 × MBC) colistin; unfilled square: 10 × MBC colistin

Low level potassium loss was detected in untreated control populations of 1 × 10^6^ cfu mL^− 1^ *E. coli* (1.3% at time zero, and 3.6% after 24 h) in this study (Fig. 1a), probably a consequence of shear forces encountered during sample centrifugation (Peterson et al. 2012) and low level osmotic lysis occurring in the hypotonic potassium-free environment (Cushnie et al. 2009). *E. coli* populations treated with inhibitory-bactericidal (0.28 mg L^−1^; 1 × MIC and 1 × MBC) and supra-bactericidal (2.8 mg l^−1^; 10 × MBC) levels of colistin, respectively, lost 5.7 and 5.9% of their total potassium content within 1 h of exposure (Fig. 1a). Potassium loss readings taken at 24 h were not much higher, just 5.9% for 1 × MIC (1 × MBC) treated cells and 11.6% for 10 × MBC treated cells.

A similar response was seen with *P. aeruginosa* (Fig. 1b). Low level potassium loss was detected in untreated control populations of 1 × 10^6^ cfu mL^−1^ *P. aeruginosa* (1.4% at time zero, and 3.7% after 24 h). Suspensions of *P. aeruginosa* treated with inhibitory-bactericidal (1.5 mg L^−1^; 1 × MIC and 1 × MBC) and supra-bactericidal (15 mg L^− 1^; 10 × MBC) levels of colistin, respectively, lost 3.8 and 4.0% of their total potassium pool within 1 h, these values remaining unchanged or almost unchanged (3.8 and 4.1%, respectively) when cells were examined at 24 h.

### Analysis of colistin-treated bacterial populations by flow cytometry

**Fig. 2:**
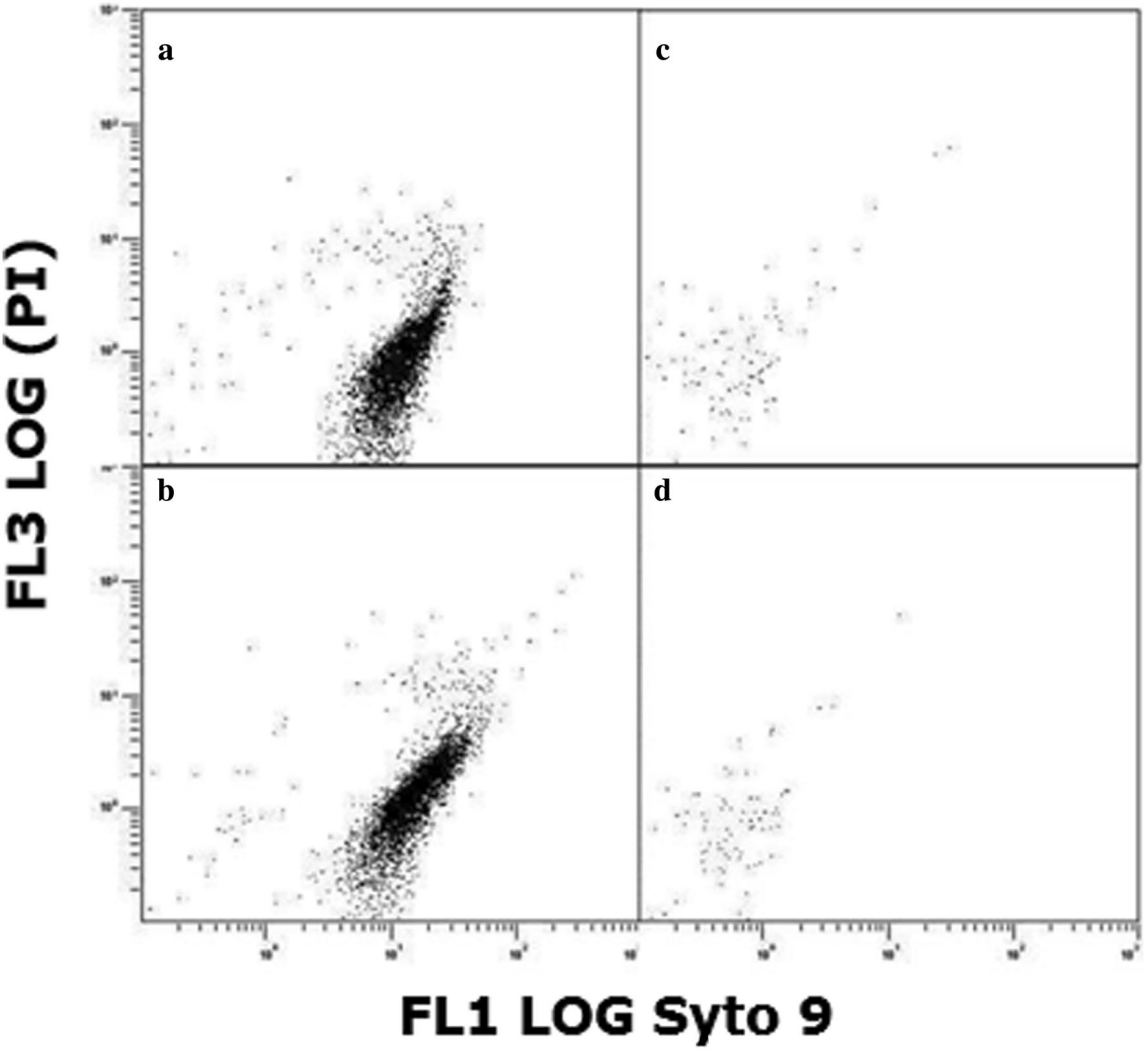
Flow cytometric analysis of populations of 1 × 10^6^ cfu mL^− 1^ *E. coli* incubated for 24 h. **a** Without colistin, **b** with 1/20 × MIC colistin, **c** with 1 × MIC (equal to 1 × MBC) colistin, and **d** with 10 × MBC colistin. FL1 LOG channel measured Syto9 fluorescence and FL3 LOG channel measured propidium iodide (PI) fluorescence

To further scrutinize the effects of colistin, we sampled and analysed by flow cytometry populations of 1 × 10^6^ cfu mL^−1^ bacteria exposed to the antibiotic for 24 h. For populations of *E. coli* treated with sub-inhibitory (1/20 × MIC) levels of colistin (Fig. 2b), there was only a slight difference compared to the untreated control (Fig. 2a). In samples of *E. coli* treated with inhibitory-bactericidal (1 × MIC and 1 × MBC) and supra-bactericidal (10 × MBC) levels of colistin, by contrast, very few events were detected in the gated population (Fig. 2c, d). The discriminator was set at a threshold of 0.2 µm such that only cells or structures larger than this and within the gate would be detected.

**Fig. 3:**
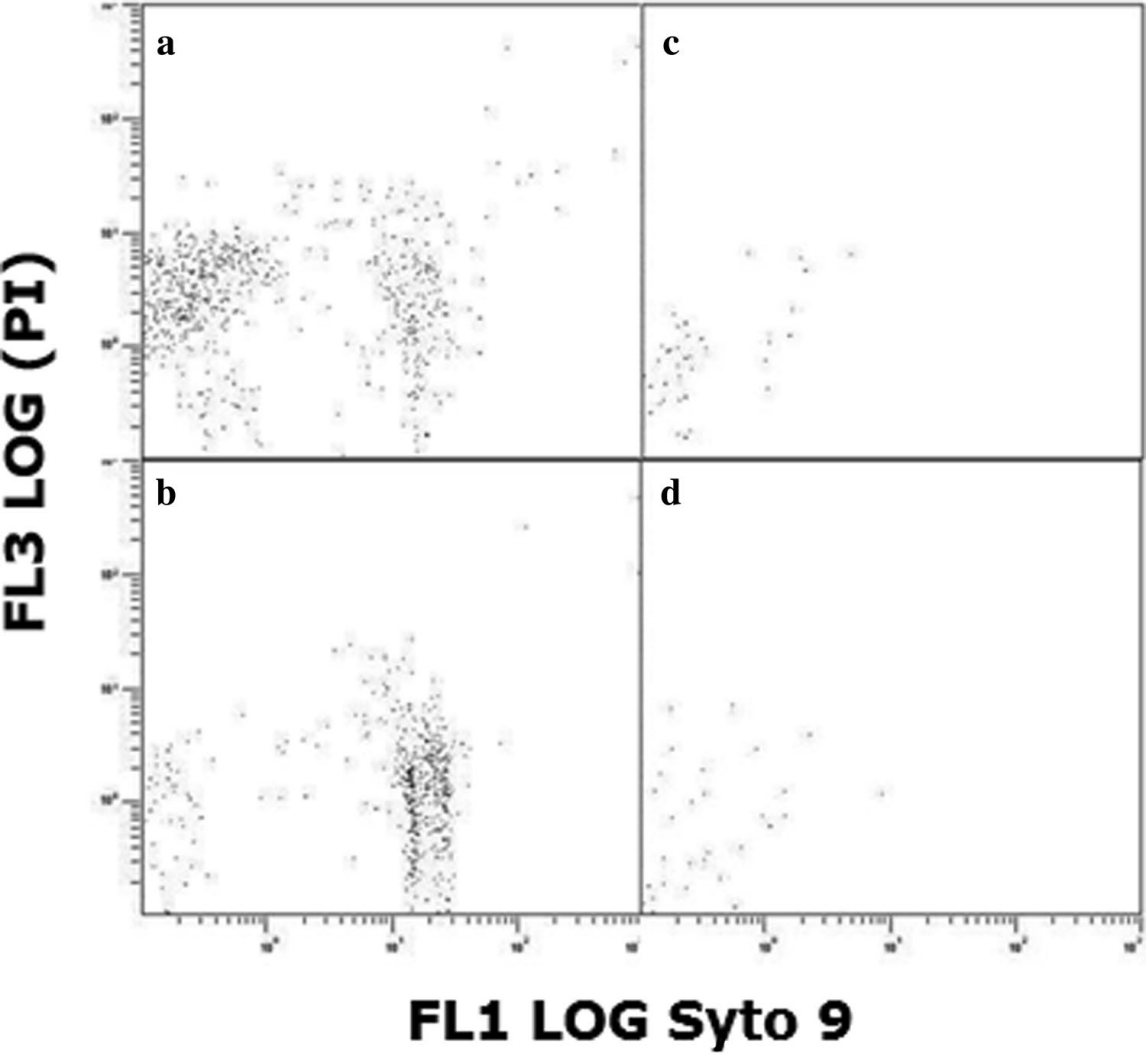
Flow cytometric analysis of populations of 1 × 10^6^ cfu mL^−1^ *P. aeruginosa* incubated for 24 h. **a** Without colistin, **b** with 1/20 × MIC colistin, **c** with 1 × MIC (equal to 1 × MBC) colistin, and **d** with 10 × MBC colistin. FL1 LOG channel measured Syto9 fluorescence and FL3 LOG channel measured propidium iodide (PI) fluorescence

The *P. aeruginosa* control culture (Fig. 3a) appeared more heterogeneous than the *E. coli* control culture (Fig. 2a), with two sub-groups apparent within the gated population. These two sub-groups probably reflect diversity of individual bacterial cell size in a normal growing population, and simply indicate that overnight *P. aeruginosa* cultures contain cells which are in two size groupings. Having established that two groups exist, examination of bacterial populations following treatment with sub-inhibitory (1/20 × MIC) colistin revealed a decrease in number of the smaller-sized cells and a slight increase in number of the larger-sized cells (Fig. 3b). In the *P. aeruginosa* cultures treated with inhibitory-bactericidal (1 × MIC and 1 × MBC) and supra-bactericidal (10 × MBC) levels of colistin, very few events were detected in the gated population (Fig. 3c, d).

### Examination of the effect of colistin treatment on bacterial cells and populations by SEM

**Fig. 4:**
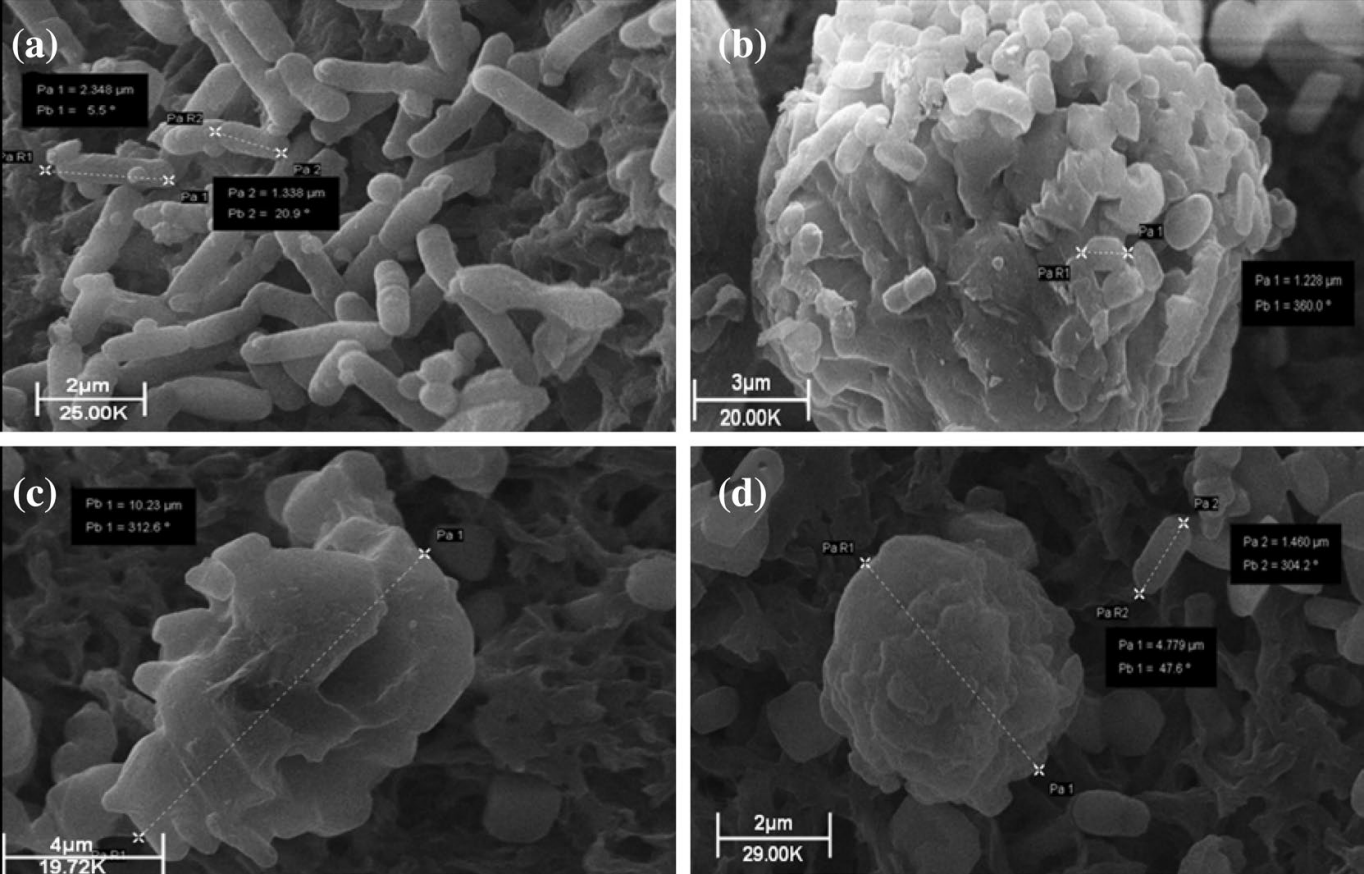
Scanning electron micrographs of populations of 1 × 10^7^ cfu mL^− 1^ *E. coli* incubated for 60 min. **a** Without colistin, **b** with 1/20 × MIC colistin, **c** with 1 × MIC (equal to 1 × MBC) colistin, and **d** with 10 × MBC colistin

Treatment of populations of 1 × 10^7^ cfu mL^−1^ *E. coli* with sub-inhibitory (0.49 mg L^−1^; 1/20 × MIC), inhibitory-bactericidal (9.8 mg L^−1^; 1 × MIC and 1 × MBC), and supra-bactericidal (98 mg l^−1^; 10 × MBC) levels of colistin triggered an immediate response. Within 60 min, profound cell aggregation was observed, these aggregates being spherical in shape and measuring 5–15 µm in diameter depending on the antibiotic concentration (Fig. 4). Aggregates remained visible in the colistin-treated populations at all concentrations [1/20 × MIC, 1 × MIC (1 × MBC), and 20 × MBC] and time-points (1, 2, 4, 8, and 24 h; data shown for 60 minutes only), although in populations treated with sub-inhibitory colistin (1/20 × MIC) the aggregates displayed more wide-ranging sizes (Fig. S1; Supplementary material). No change in cell length was detected at any colistin concentration or any time-point with *E. coli*. No blebbing of the bacterial cell surface was detected at any concentration or time-point with *E. coli* either.

**Fig. 5:**
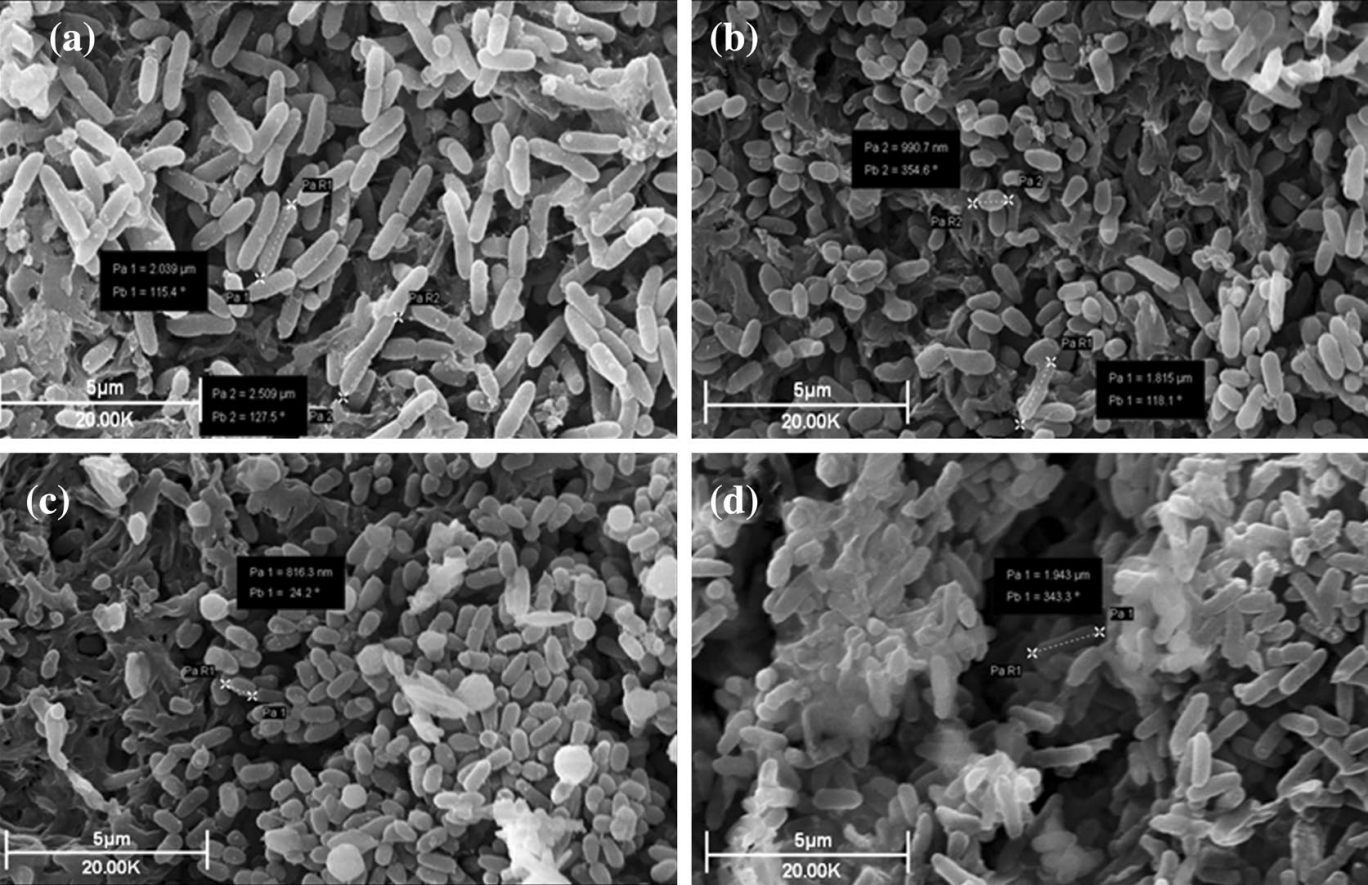
Scanning electron micrographs of populations of 1 × 10^7^ cfu mL^− 1^ *P. aeruginosa* incubated for 60 min. **a** Without colistin, **b** with 1/20 × MIC colistin, **c** with 1 × MIC (equal to 1 × MBC) colistin, and **d** with 10 × MBC colistin

For populations of 1 × 10^7^ cfu mL^−1^ *P. aeruginosa* treated with sub-inhibitory (0.25 mg l^−1^; 1/20 × MIC), inhibitory-bactericidal (4.9 mg L^−1^; 1 × MIC and 1 × MBC), and supra-bactericidal (49 mg l^−1^; 10 × MBC) levels of colistin, the morphological changes observed were both concentration- and time-dependent. After 60 min, a decrease in the length of individual cells was detected in populations treated with sub-inhibitory (1/20 × MIC) and inhibitory-bactericidal (1 × MIC and 1 × MBC) levels of colistin (Fig. 5b and c). Compared to control cells which were 2.0–2.5 µm in length, cells treated with sub-inhibitory levels of colistin were just 0.9–1.8 µm in length, and cells treated with inhibitory-bactericidal levels of colistin were only ~ 0.8 µm in length. Aggregation was only detected at the highest colistin concentration (10 × MBC) at 60 min (Fig. 5d), but became apparent for the other two concentrations [1/20 × MIC and 1 × MIC (1 × MBC)] by 2 h (data not shown). This aggregation initially manifested as layers of bacteria interlinked by strands of extracellular material, with spherical clumps not detectable until 8 h and remaining extremely sparse until 24 h (Fig. S2c and S2d; Supplementary material). Blebbing of the bacterial cell surface was detected from 8 h onwards (data not shown).

## Discussion

The polymyxin antibiotic colistin has recently been resurrected for the treatment of infections with extensively drug-resistant Gram-negative bacteria. We investigated the impact of a range of colistin concentrations upon *E. coli* and *P. aeruginosa* populations to obtain a clearer understanding of how this antibiotic functions. To determine if colistin compromises cytoplasmic membrane integrity as part of its antibacterial mechanism of action, we examined populations of *E. coli* and *P. aeruginosa* for loss of the intracellular solute potassium. The data obtained show that, even after 24 h treatment with supra-bactericidal (10 × MBC) levels of colistin, *E. coli* populations lost less than 12% of their total potassium pool and *P. aeruginosa* populations lost less than 5%. Genuinely cytoplasmic membrane-active agents such as protegrin and cathelicidin have, by comparison, been shown to induce 90–100% potassium loss in similar studies with *E. coli* in less than 1 h (Orlov et al. 2002; Bolintineanu et al. 2010). Overall, the fact that colistin-treated populations retain more than 85% of their total potassium pool confirms the cytoplasmic membrane remains intact, if not functional, in most of the treated cells. Results presented here correlate well with two previous studies, the first by Zhang et al. (2000) which showed that colistin exerts a lethal effect on *P. aeruginosa* without affecting cytoplasmic membrane proton motive force, and the second by Alhanout and colleagues (2010), which showed that 4 h treatment with 4 × MIC colistin led to *E. coli* and *P. aeruginosa* populations losing just 4–5% of their total intracellular ATP. Taken together, these findings argue against the longstanding and widely cited hypothesis that colistin kills bacterial cells by puncturing the cytoplasmic membrane.

In the next stage of the study, antibiotic-treated populations of bacteria were examined by flow cytometry, using Syto9 and PI fluorescent dyes to identify intact cells of viable and non-viable bacteria, respectively. For *E. coli*, treatment with inhibitory-bactericidal (1 × MIC and 1 × MBC) or supra-bactericidal (10 × MBC) levels of colistin resulted in a near-complete loss of signal from the gated population (Fig. 2c, d). In the absence of large-scale potassium loss (Fig. 1a), this result cannot be due to colistin-induced cell lysis. Rather, colistin must be altering the size or arrangement of cells in such a way that they are not being detected. With *P. aeruginosa*, flow cytometry revealed the presence of two slightly differently sized sub-populations in overnight cultures (Fig. 3a), the number of smaller-sized cells decreasing following treatment with sub-inhibitory (1/20 × MIC) levels of colistin (Fig. 3b). This pattern of results might suggest that a cell cycle event subsequent to septation and cell separation renders bacteria susceptible to colistin-induced cell lysis. Perhaps more likely though, given the accompanying increase in number of larger-sized cells (Fig. 3b), colistin is inhibiting cell separation or causing cell aggregation. As with *E. coli*, treatment of *P. aeruginosa* with inhibitory-bactericidal (1 × MIC and 1 × MBC) or supra-bactericidal (10 × MBC) levels of colistin resulted in a near-complete loss of signal (Fig. 3c, d), suggesting colistin modifies cell size or arrangement in such a way that they are no longer detected.

SEM studies were performed next to visualize directly the impact of colistin upon whole populations of *E. coli* and *P. aeruginosa*. With *E. coli*, the observation of colistin-induced aggregation of bacterial cells (Fig. 4 and Fig. S1) may explain the small number of events detected during flow cytometry (Fig. 2c, d) as aggregation would greatly reduce the number of individual cells present. Colistin-induced aggregation of bacteria has not, to our knowledge, been reported previously, but may be more readily detectable by SEM than TEM or atomic force microscopy. Aggregation similar to this has been reported in bacteria treated with other cationic antimicrobial peptides (O’Driscoll et al. 2013), but the reason for this population-level change is not clear. It may be a direct effect of the antibiotic, for example colistin molecules inserting into the outer membrane of cells and promoting intercellular attachment in a manner comparable to that observed with liposomes (Wallace et al. 2012). Alternatively, it may be a coordinated response on the part of the bacterial population to minimize antibiotic exposure. Young suggests the ability of bacteria to form aggregates is an evolutionary response to predation by protozoa (Young 2007) and, as observed with bacterial biofilms (Costerton et al. 1999), aggregation offers protection from antibiotic treatment also. Unlike previous studies with colistin-treated *E. coli* (Koike et al. 1969), we did not observe any blebbing of the bacterial outer membrane, but this is more readily detectable by TEM than SEM (Cushnie et al. 2016).

With *P. aeruginosa*, sub-inhibitory (1/20 × MIC) and inhibitory-bactericidal (1 × MIC and 1 × MBC) levels of the antibiotic both caused a decrease in bacterial cell length (Fig. 5 and Fig. S2) within 1 h. These results correlate well with an atomic force microscopy study by Mortensen et al., which found that sub-MBC colistin-treated cells of *P. aeruginosa* also exhibited reduced cell length (Mortensen et al. 2009). These observations suggest that colistin treatment, either directly or indirectly, leads to inhibition of lateral cell wall formation. Possible targets include the cytoskeletal Mre system (proteins MreB, MreC and MreD) or enzyme PBP2, both of which are essential for lateral peptidoglycan synthesis and elongation of the bacterial cell (Osborn and Rothfield 2007; Varma and Young 2009). Both the Mre system and PBP2 are anchored to the cytoplasmic membrane (Adachi et al. 1987; Osborn and Rothfield 2007), and would be vulnerable to any colistin-induced conformational changes taking place at this locale. Colistin-induced aggregation of *P. aeruginosa* cells, whilst slightly different in appearance to that visualised with *E. coli*, was observed (Fig. 5 and Fig. S2) and may again account for the small number of events detected during flow cytometry (Fig. 3c, d). Blebbing of colistin-treated *P. aeruginosa* was also detected in this study, an observation reported previously by Koike et al. (1969) and Alhanout et al. (2010). This ultrastructural change, because it is induced by multiple membrane-active agents including chlorhexidine and EDTA, is considered an indicator of possible outer membrane disruption (Cushnie et al. 2016).

When the SEM results for *E. coli* and *P. aeruginosa* are viewed conjointly, it is clear there are both similarities and differences in how colistin affects these bacteria. Filamentation was conspicuous by its absence in both species. Filamentation is a hallmark of DNA damage, induced via the SOS response by a diverse range of physical and chemical agents including UV, cosmomycin D, metronidazole, mitomycin C, the fluoroquinolones, novobiocin, and zidovudine (Cushnie et al. 2016). The failure of colistin to induce filamentation at any concentration or time-point in either species argues strongly against the hypothesis that colistin kills bacterial cells through DNA damage or disruption of DNA synthesis. Colistin-induced cell aggregation was, conversely, detected in both species. Whilst it is not clear how, or even if, this aggregation relates to colistin mechanism of action, it could have important implications for diagnostic laboratories. Colistin-induced clumping of cells would reduce colony forming unit (cfu) numbers of bacteria in MBC and time-kill assays, resulting in artificially low MBC values for the antibiotic. Colistin-induced cell aggregation could also complicate how the course of infections is monitored because, in patients receiving this antibiotic, false-negative blood and urine cultures would be more likely to occur. Moving on to differences in how colistin affects the bacteria, the most noticeable of these was the reduction in cell size observed with *P. aeruginosa* but not *E. coli*. This might be due to slight variations in the cell envelope composition or PBP2/Mre protein conformation of the two species. Ultimately, this and other observed interspecies variations probably reflect indirect or peripheral aspects of colistin’s mechanism of action as both bacterial species are susceptible to the antibiotic.

In conclusion, our study has shown that colistin induces minimal potassium loss and no filamentation in populations of *E. coli* or *P. aeruginosa*, findings which indicate the antibiotic does not exert its antibacterial effect by perforating the cytoplasmic membrane or disrupting DNA synthesis. Future studies should therefore focus on exploring the three remaining hypotheses, namely that colistin kills bacterial cells by (1) inducing phospholipid exchange between the outer and cytoplasmic membranes, (2) inhibiting respiratory enzymes, and/or (iii) inducing reactive oxygen species formation. Colistin-induced aggregation of bacterial cells hindered our attempt to study this antibiotic’s effects by flow cytometry, but represents an interesting observation in itself, one with implications for the interpretation of MBC, time-kill, and other microbiology assay results.

## Electronic supplementary material

Below is the link to the electronic supplementary material.

Supplementary material 1 (DOCX 946 KB)

Supplementary material 2 (DOCX 1063 KB)
