# Supplementary material for: Colistin causes profound morphological alteration but minimal cytoplasmic membrane perforation in populations of Escherichia coli and Pseudomonas aeruginosa
Source: Arch Microbiol. 2018 Feb 8;200(5):793–802. doi: 10.1007/s00203-018-1485-3 (PMC6004271; doi:10.1007/s00203-018-1485-3)
Supplement: Supplementary file 1 — Supplementary material 1 (DOCX 946 KB) [file 203_2018_1485_MOESM1_ESM.docx]

**Colistin causes profound morphological alteration but minimal cytoplasmic membrane perforation in populations of *Escherichia coli* and *Pseudomonas aeruginosa***

**Noëlle H. O’Driscoll, T.P. Tim Cushnie, Kerr H. Matthews, Andrew J. Lamb**


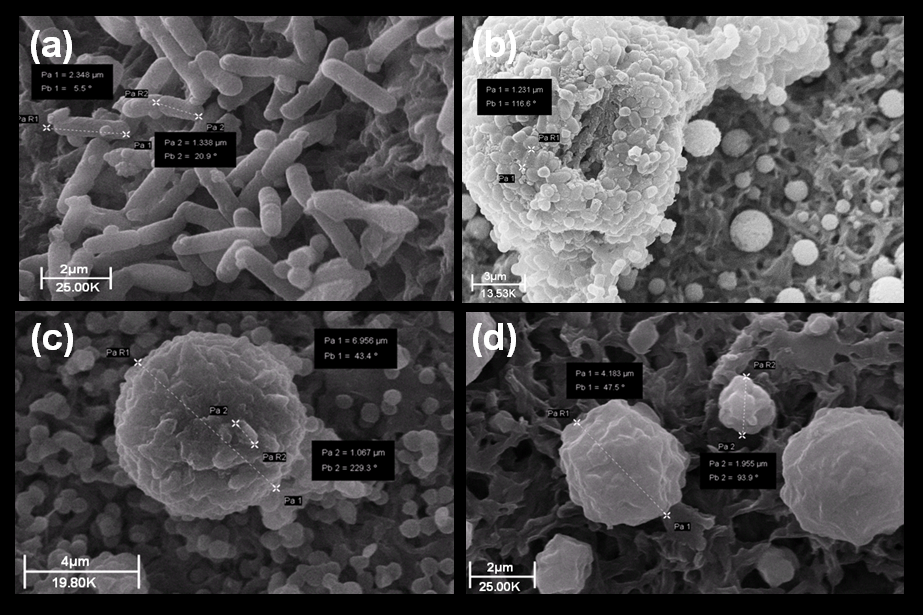


**Fig. S1** Scanning electron micrographs of populations of 1 x10^7^ cfu ml^-1^ *E. coli* incubated for 24 h (a) without colistin, (b) with 1/20xMIC colistin, (c) with 1xMIC (equal to 1xMBC) colistin, and (d) with10xMBC colistin.
