# Supplementary material for: Colistin causes profound morphological alteration but minimal cytoplasmic membrane perforation in populations of Escherichia coli and Pseudomonas aeruginosa
Source: Arch Microbiol. 2018 Feb 8;200(5):793–802. doi: 10.1007/s00203-018-1485-3 (PMC6004271; doi:10.1007/s00203-018-1485-3)
Supplement: Supplementary file 2 — Supplementary material 2 (DOCX 1063 KB) [file 203_2018_1485_MOESM2_ESM.docx]

**Colistin causes profound morphological alteration but minimal cytoplasmic membrane perforation in populations of *Escherichia coli* and *Pseudomonas aeruginosa***

**Noëlle H. O’Driscoll, T.P. Tim Cushnie, Kerr H. Matthews, Andrew J. Lamb**


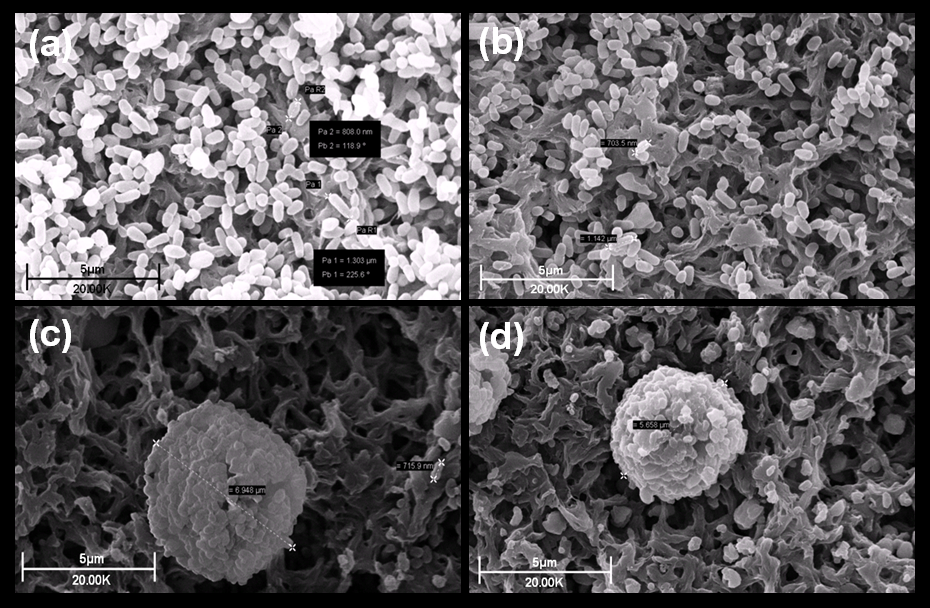


**Fig. S2** Scanning electron micrographs of populations of 1 x10^7^ cfu ml^-1^ *P. aeruginosa* incubated for 24 h (a) without colistin, (b) with 1/20xMIC colistin, (c) with 1xMIC (equal to 1xMBC) colistin, and (d) with 10xMBC colistin.
